# Label-Free Electrochemical Interleukin‑6 Sensor Exploiting rGO-Ti_3_C_2_T_*x*_ MXene Nanocomposites

**DOI:** 10.1021/acsami.5c06701

**Published:** 2025-07-24

**Authors:** Rohit Gupta, Ashish Kalkal, Priya Mandal, Diptiranjan Paital, David Brealey, Manish K. Tiwari

**Affiliations:** † Nanoengineered Systems Laboratory, UCL Mechanical Engineering, 4919 University College London, London WC1E 7JE, U.K.; ‡ UCL Hawkes Institute, 4919 University College London, London W1W 7TS, U.K.; § Department of Physics, 4616 King’s College London, London WC2R 2LS, U.K.; ∥ Division of Critical Care, 8964 University College London Hospitals, London NW1 2BU, U.K.; ⊥ NIHR University College London Hospitals Biomedical Research Centre, London NW1 2BU, U.K.; # Manufacturing Futures Laboratory, 4919 University College London, London E20 2AE, U.K.

**Keywords:** immunosensor, interleukin-6, MXene, graphene oxide, point-of-care diagnostics, electrochemical simulation, thin-layer diffusion

## Abstract

This work introduces a novel, rapid, label-free, affinity-enabled electrochemical sensor for the detection of interleukin-6 (IL-6), a critical proinflammatory cytokine associated with severe conditions like sepsis and COVID-19. Unlike conventional approaches, this platform leverages an innovative biofunctional nanocomposite of Ti_3_C_2_T_*x*_ MXene, tetraethylene pentaamine-functionalized reduced graphene oxide (TEPA-rGO), and Nafion, functionalized with anti-IL-6 antibodies, integrated into a carbon-based screen-printed three-electrode chip. The system achieves unprecedented sensitivity in IL-6 quantification, with a single-digit pg/mL detection limit and a broad range of 3–1000 pg/mL using ∼5 μL of serum. The sensor design is uniquely enhanced through the introduction of a genetic algorithm-based thin-layer diffusion model, which optimizes critical, previously unknown electrochemical transport parameters, including diffusion coefficient, rate constant, charge transfer coefficient, and electrochemically active surface area. This approach represents a significant advancement in biosensor modeling and performance tuning. The sensor demonstrates exceptional selectivity (signal-to-noise ratio ∼ 6.9) against relevant interferents (e.g., sepsis-related antigens, small molecules, electroactive compounds), retains operational stability for a month, and offers a sample-to-answer time of ∼15 min (i.e., up to 12 times faster than traditional ELISA), while maintaining comparable sensitivity. Detailed morphological, topographical, and chemical analyses validate the structural and functional integrity of the TEPA-rGO/MXene/Nafion nanocomposite. By combining cutting-edge nanomaterials with advanced computational modeling, this IL-6 sensor sets a new benchmark for rapid, precise cytokine detection, offering transformative potential for early disease diagnosis and prognosis.

## Graphical abstract

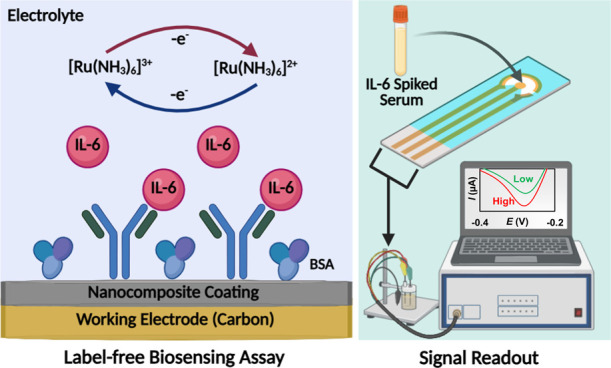

## 1 Introduction

Profiling proinflammatory cytokines like interleukin-6 (IL-6) in body fluids offers critical insights into immune responses related to conditions such as sepsis,^1^ myocardial infarction,^2^ COVID-19,^3^ and neurodegenerative disorders.^4^ Monitoring IL-6 in the whole blood, serum, or plasma is particularly valuable for assessing the inflammation severity, for prognostication,^5^ and for identifying subphenotypes^6^ with the potential to guide novel therapies. In healthy adults, IL-6 levels typically range from 1 to 5 pg/mL, whereas in older adults, levels are between 5 and 20 pg/mL.^7^ Elevated IL-6 concentrations correlate with inflammation, underscoring the importance of rapid, accurate diagnostics in point-of-care (POC) settings.

Traditional IL-6 quantification methods, including flow cytometry, Western blot, quantitative polymerase chain reaction, and enzyme-linked immunosorbent assay (ELISA),^8^ provide high sensitivity and specificity but are unsuitable for POC applications due to the large sample volume (∼10^2^–10^3^ μL), complex multistep protocols, and lengthy processing times.^9^ In contrast, electrochemical biosensors (EBs) for IL-6 detection offer advantages such as lower sample volumes (1–10 μL), faster turnaround times (<30 min), high sensitivity and selectivity, and potential for miniaturization, making them ideal for POC diagnostics.^10^

There are two main types of antibody-based EBs for IL-6 detection: the labeled-sandwich assay and the direct label-free assay. The labeled-sandwich EBs are similar to sandwich ELISA, where the IL-6 biomarker is captured between two antibodies, with the detection antibody tagged with enzymes (e.g., horseradish peroxidase) or redox labels (e.g., ferrocene) that generate electrical signals upon analyte binding.^8^ Conversely, label-free EBs detect changes in the electrical properties of the biosensing interface due to antigen–antibody interactions, with the degree of interaction affecting charge transfer resistance and Faradaic peak current across the electrolyte–electrode interface, typically measured using redox cycling of [Fe­(CN)_6_]^3–/4–^ or [Ru­(NH_3_)_6_]^2+/3+^.^11^ While sandwich EBs offer signal amplification, label-free EBs are favored for their rapid, single-step detection mechanism, making them more suitable for POC applications.

Developing suitable nanomaterials for label-free EB interfaces is an emerging research area that requires trade-offs among electrical conductivity, redox species diffusion, biomolecule immobilization, and sensor contamination (or fouling). Various nanomaterials have been utilized to develop label-free IL-6 sensors, including gold (Au)-based nanomaterials (e.g., Au microneedles,^12^ Au nanoparticles,^13–16^ Au nanowires^17^), conductive polymer films (polypyrrole,^18,19^ PEDOT:PSS^20^), carbon-based nanomaterials (e.g., multi- and single-walled carbon nanotubes,^14^ ^,^ ^21^ biochar,^22^ graphene oxide^23,24^ ), and nanostructured metallic oxides (e.g., ZnO film,^25^ α-Fe_2_O_3_ nanorod^26^); many of these approaches face critical limitations. These include suboptimal charge transfer, limited surface area, poor wetting properties, and insufficient stability, all of which hinder their performance as label-free EB interfaces.

In recent years, graphene derivatives, particularly reduced graphene oxide (rGO), have garnered significant attention due to their Au-like charge transfer properties (i.e., high Faradaic current), tunable functional groups, and scalability. However, rGO has limitations, including inadequate charge storage capacity and hydrophilicity, which can reduce the performance of label-free EBs. In light of this, titanium carbide (Ti_3_C_2_T_*x*_) MXene has emerged as a promising functional 2D material, offering higher electrical conductivity, larger surface area, better hydrophilicity, areal capacitance, and structural integrity.^27^ Incorporating MXene can enhance the electroactive area and functional group availability, thereby increasing both the Faradaic and capacitive currents and biomolecule loading. Consequently, the present study aims to develop a nanocomposite comprising rGO and Ti_3_C_2_T_*x*_ MXene to optimize both Faradaic and capacitive currents.

To facilitate strong covalent interactions between the rGO-MXene nanocomposite and biomolecules (i.e., antibodies), this study employs tetraethylene pentaamine-functionalized rGO (TEPA-rGO). In addition to high electrical conductivity and a favorable area-to-volume ratio, the nanomaterial films used in biosensors must be reproducible and resistant to leaching and fouling. To address these needs, Nafion, a sulfonated fluoropolymer, is used.^28^ The TEPA-rGO/MXene/Nafion nanocomposite can be quickly drop-cast onto an electrochemical platform, and functionalizing it with antibodies enables a specific interaction with IL-6 in serum samples.

Understanding quasi-reversible redox parameters, such as material-specific diffusion characteristics (e.g., diffusivity (*D*) and electrochemically active surface area (*ECSA*)) and rate-determining kinetics (rate constant (*k*_0_) and charge transfer coefficient (α)), is crucial for label-free EBs as they provide insights into the biosensing mechanism. However, these parameters are often unknown for existing biosensor interfaces.

To address this gap, the study integrates a one-dimensional (1D) spatiotemporal thin-layer diffusion model^29^ with a genetic algorithm (GA)-based optimization framework, enabling iterative refinement of these electrochemical parameters by aligning the experimental and simulated cyclic voltammetry (CV) responses. The data-driven mechanistic model not only elucidates the redox kinetics of the TEPA-rGO/MXene/Nafion nanocomposite but also facilitates rapid designing optimization by leveraging the porous nanocomposite’s ability to enhance thin-layer diffusion and electron tunneling.^29^

Building on these insights, a label-free IL-6 biosensor was developed, offering clinically relevant sensitivity and enhanced specificity. Comprehensive morphological, chemical, and electrochemical characterizations were performed to better understand the biosensing mechanism. The sensor’s single-step protocol achieves a detection limit of 2.1 pg/mL and operates within a sensing range of 3–1000 pg/mL in serum samples, with a rapid turnaround time of approximately 15 min. Furthermore, the biosensor exhibits improved stability and enhanced selectivity against other sepsis-related antigens (Supporting Information), positioning it as a highly promising tool for IL-6 detection in POC diagnostics.

## 2 Materials and Methods

### 2.1 Reagents

Delaminated Ti_3_C_2_T_*x*_ MXene nanoflakes were procured from Nanoplexus Ltd. Screen-printed carbon electrode (SPCE) chips (DropSens 110) containing three electrodes were purchased from Metrohm Ltd. (working electrode (WE): carbon; counter electrode (CE): carbon; and reference electrode (RE): silver) (Figure 1). TEPA-rGO, Nafion 117 (5% solution), hexaamineruthenium­(III) chloride, and bovine serum albumin (BSA) were purchased from Sigma-Aldrich. 1-Ethyl-3-(3-(dimethylamino)­propyl) carbodiimide hydrochloride (EDC), *N*-hydroxysuccinimide (NHS), 2-(*N*-morpholino)­ethanesulfonic acid (MES, pH 6.2), potassium chloride (KCl), phosphate buffer saline (PBS) tablets, human AB serum (hemoglobin content <26 mg/dL), human IL-6 protein (CHC1263), and human anti-IL-6 antibody (CHC1263) were procured from Thermo Fisher Scientific. Unless specified, all of the reagents were prepared using 10 mM PBS in deionized (DI) water (pH ∼ 7.2).

**Figure 1:**
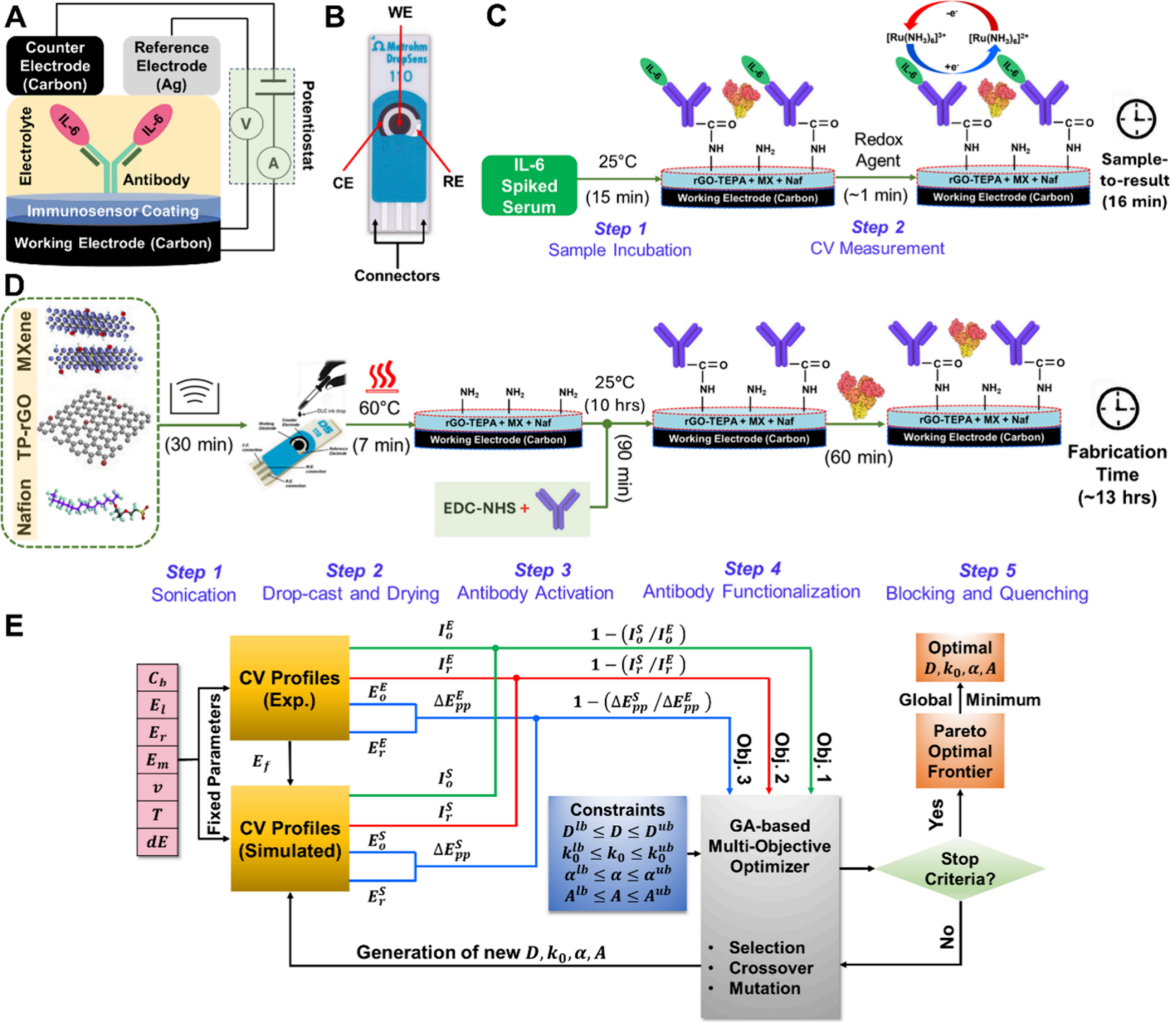
Overview of the IL-6 EB platform. (A) Schematic illustration of the ionic circuit representing the three-electrode sensing mechanism. (B) Image of the DropSens SPCE chip comprising the WE, CE, and RE. (C) Label-free detection strategy for IL-6 spiked into human serum. (D) Step-by-step fabrication protocol for the IL-6 biosensor. (E) Mechanistic model integrated with a GA for extracting unknown electrochemical parameters to elucidate the biosensor’s response.

### 2.2 Nanocomposite Synthesis, Sensor Fabrication, and Sample Detection

Ti_3_C_2_T_*x*_ MXene nanoflakes and TEPA-rGO were dispersed in DI water via probe sonication for 3 h, resulting in stock concentrations of 4 and 10 mg/mL, respectively. A series of nanocomposite suspensions were then prepared with varying concentrations of MXene (0.25, 0.5, 1, 1.5, and 2 mg/mL), TEPA-rGO (0.25, 1, 1.5, 2, and 3 mg/mL), and Nafion (0.25, 0.5, 0.75, 1, and 1.5%). To ensure uniform integration of MXene and TEPA-rGO into the Nafion matrix, the TEPA-rGO/MXene/Nafion nanocomposite was bath-sonicated for 30 min with a 1 s ON/OFF pulse. Approximately 10 μL of the prepared nanocomposite was then drop-cast onto the WE of an SPCE and dried in a hot air oven at 60 °C for 7 min. The chips were subsequently washed with PBS while agitated at 400 rpm to remove any excess nanomaterial, followed by drying under a gentle nitrogen (N_2_) stream.

For affinity-enabled IL-6 sensing, anti-IL-6 antibodies were immobilized on the nanocomposite-coated electrodes using carbodiimide heterobifunctional cross-linking (i.e., EDC-NHS) chemistry.^17^ Specifically, a ternary mixture containing 400 mM EDC, 200 mM NHS, and 50 μg/mL human anti-IL-6 antibody was prepared in 50 mM MES buffer (pH ∼ 6.2) and placed on an orbital shaker at room temperature for 90 min to activate the antibody’s carboxyl groups. A 6 μL aliquot of this mixture was then applied to the nanocomposite-modified WE, followed by overnight incubation at room temperature in a humidified chamber to facilitate the formation of amide linkages between the antibody and nanocomposite. To optimize and evaluate the minimum capture antibody (cAb) density on the surface, two additional cAb concentrations (100 and 200 μg/mL) were also tested. The chips were subsequently incubated with 2.5 wt % (25 mg/mL) BSA for 1 h to block any unreacted sites on the WE. Each fabrication step was followed by washing with approximately 3 mL of 10 mM PBS and drying with N_2_. The antibody-functionalized IL-6 sensing chips were stored at 4 °C until further use.

Testing samples were prepared by spiking a known amount of IL-6 protein (between 1 and 1000 pg/mL) in human AB serum. Next, 5 μL of this sample was applied to the WE area and incubated in a humidified chamber for various incubation times (5, 15, 30, and 60 min). No additional sample preparation steps beyond serum extraction would be required for the practical deployment of the sensor. After incubation, the chip was washed once with PBS to remove any unbound IL-6 and dried under N_2_. The SPCE chip was then studied using CV in a 100 μL puddle containing 10 mM [Ru­(NH_3_)_6_]­Cl_3_ and 100 mM KCl dissolved in PBS, with a scan rate (*v*) of 100 mV/s (Figure 1).

### 2.3 Material Characterization

CV was conducted using the IviumStat electrochemical workstation within a potential range from −0.6 to 0.1 V, employing 10 mV potential steps with *v* = 100 mV/s. Cyclic voltammograms were obtained with a 100 μL droplet containing 10 mM [Ru­(NH_3_)_6_]^3+^ and 100 mM KCl dissolved in PBS, with the average response from three CV cycles reported. The recorded current responses (in μA) were converted to current density (μA/mm^2^) based on the geometric area (*A*_g_) of the WE, which is 12.56 mm^2^.

Contact angle (CA) measurements were performed by placing an ∼4 μL droplet onto the WE, with each measurement repeated three times. Image analysis and CA extraction were conducted using a custom-developed MATLAB script. Surface profiles of the functionalized WE were derived through atomic force microscope imaging in the noncontact tapping mode (using a Bruker MultiMode 8-HR system), utilizing tips with a curvature radius of ∼20 nm. The average (*R*_a_) and root mean-squared roughness (*R*_RMS_) values were determined using NanoScope 2.0 software.

Morphological and elemental characterization of the TEPA-rGO/MXene nanocomposite was conducted using a Zeiss EVO LS15 scanning electron microscope (SEM) equipped with an Oxford Instruments energy-dispersive X-ray spectroscopy (EDS) system. The nanocomposite was deposited onto a cleaned glass slide and coated with a ∼10 nm gold layer via sputtering. SEM imaging was performed using an in-lens secondary electron detector at an accelerating voltage of 20 kV.

Chemical state analysis of the constituent elements was conducted by using a Kα X-ray photoelectron spectroscopy (XPS) system (Thermo Fisher Scientific Nexsa) equipped with a monochromatic Al Kα X-ray source. The X-ray beam spot size was set to 400 μm^2^. High-resolution spectra were recorded with a binding energy step size of 0.1 eV, with each spectrum subjected to up to 20 scans. Spectral deconvolution was performed using CasaXPS software and applying the Shirley background correction method. All spectral fittings were carried out with a relative fitting error maintained below 5% to ensure the reliability and accuracy of the results.

The chemical composition of the TEPA-rGO/MXene nanocomposite, both before and after anti-IL-6 antibody functionalization, was further analyzed by using a Nicolet iS50 Fourier-transform infrared (FTIR) spectrometer. Transmittance spectra of powdered samples were recorded over the wavenumber range of 600–3000 cm^–1^, averaged over 32 scans. FTIR spectra were processed using Origin 2024b software, employing nonlinear baseline correction and Savitzky–Golay smoothing with a 100-point window to enhance spectral clarity and resolution.

### 2.4 Data-Driven Mechanistic Model

The redox kinetics of SPCE chips modified with different IL-6 EB interfaces were analyzed using a 1D thin-layer diffusion model, simulating CV characteristics. To determine material-specific diffusion and kinetic parameters (e.g., *D*, *ECSA, k*_0_, α) that are unknown for a biosensor, a framework is developed.^29^ This framework integrates a spatiotemporal thin-layer diffusion model with GA-based optimization to iteratively estimate these parameters by aligning the experimental and simulated CV responses. The 1D diffusion of [Ru­(NH3)_6_]^3+/2+^ perpendicular to the electrode is described by Fick’s law as

$$\frac{\partial c(z,t)}{\partial t}=D\frac{\partial^{2}c(z,t)}{\partial z^{2}}$$ 1

where *c*(*z*, *t*) is the molar concentration of [Ru­(NH_3_)_6_]^3+^, *t* is time (s), *z* is the perpendicular distance measured from the electrode surfaces (in cm), and *D* is the apparent diffusion coefficient (in cm^2^ s^–1^) of the redox couple [Ru­(NH_3_)_6_]^3+^. Solving eq 1 requires the electrode boundary condition (*t* > 0, *z* = 0), the far-field boundary condition (*t* > 0, *z* = *L*), and the initial condition over the entire domain (*t* = 0, ∀ *z*), given by eqs 2–4, respectively.

$$\frac{\partial c}{\partial z}{|}_{z=0,t>0}=\frac{k_{0}}{D}\left[c{|}_{z=0}\exp\left\{-\frac{\alpha F(E(t)-E_{f}^{0})}{RT}\right\}-(c_{b}-c{|}_{z=0})\exp\left\{\frac{\left(1-\alpha )F(E(t)-E_{f}^{0}\right)}{RT}\right\}\right]$$ 2

$$c{|}_{z=L,t>0}=c_{b}$$ 3

$$c{|}_{t=0,\forall z}=c_{b}$$ 4

Equation 2 is the Butler–Volmer equation for the single-electron transfer redox reaction where the Faraday constant *F* = 96485 A s/mol, universal constant *R* = 8.314 J/mol K, *T* = 298 K is the room temperature, *c*_b_ is the bulk concentration of [Ru­(NH_3_)_6_]^3+^, *E*_f_^0^ is the reference potential for the redox couple, and *E*(*t*) is the instantaneous linear potential waveform driving the CV experiment. As per a previous recommendation,^30^ the maximum diffusion domain length is specified as $L∼6\sqrt{(2D|E_{R}-E_{L}|)/v}$ , where *E*_R_ and *E*_L_ are the two extreme potentials for CV, and *v* is the scan rate. The diffusion current is evaluated as

$$i(t)=\mathrm{FAD}\frac{\partial c}{\partial z}{|}_{z=0,t>0}$$ 5

Equations 1–5 are discretized using a finite volume method (FVM) with 500 equally spaced grid points (see the Supporting Information for grid independence study) and the implicit time marching scheme. The resulting set of linear equations is solved using a tridiagonal matrix algorithm, determining the spatiotemporal concentration profiles and time-varying current responses.^29^ Key features, oxidation peak current (*I*_ox_^sim^), reduction peak current (*I*_red_^sim^), and potential separation between oxidation and reduction peaks (*E*_pp_^sim^) are extracted from the simulated CV. Corresponding features (*I*_ox_^exp^, *I*_red_^exp^, and *E*_pp_^exp^) are obtained from the experimental CV profiles. These two sets of features are used to evaluate the percentage of relative error between experimental and simulated *I*_ox_, *I*_red_, and *E*_pp_. The relative errors between experimental and simulated values are calculated as Δ*I*_ox_ = (*I*_ox_^exp^ – *I*_ox_^sim^)/*I*_ox_^exp^, Δ*I*_red_ = 100 × (*I*_red_^exp^ – *I*_red_^sim^)/*I*_red_^exp^, and Δ*E*_pp_ = 100 × (*E*_pp_^exp^ – *E*_pp_^sim^)/*E*_pp_^exp^ and minimized using GA. The optimization adjusts parameters *k*_0_, *D*, α, and *ECSA*, providing electron transport characteristics of the biosensing interfaces. The multiobjective error minimization problem is formally defined as follows:

$$\begin{array}{l} \min_{D,k_{0},\alpha ,\mathrm{ECSA}}[\Delta I_{\mathrm{ox}},\Delta I_{\mathrm{red}},\Delta E_{\mathrm{pp}}]\\ s.t.\;D^{lb}\leq D\leq D^{ub}\\ \;\;\;\;\;\;k_{0}^{lb}\leq k_{0}\leq k_{0}^{ub}\\ \;\;\;\;\;\;\alpha^{lb}\leq \alpha \leq \alpha^{ub}\\ \;\;\;\;\;\;{\mathrm{ECSA}}^{lb}\leq \mathrm{ECSA}\leq {\mathrm{ECSA}}^{ub} \end{array}$$ 6

After the Pareto front optimization converges, the point closest to the origin in the three-dimensional error space is selected as the elitist solution for *k*_0_, *D*, α, and *ECSA*. The process is repeated up to three times for each biosensing interface for accurate parameter estimation. A summary of the inverse parameter learning algorithm is provided in Figure 1E, with further details in the Supporting Information and the source code provided in the repository (https://github.com/rohitgupta280195/InverseLearningBiosensorParameters.git).

## 3 Results and Discussion

### 3.1 Morphological and Chemical Characterization of the Nanocomposite

The morphology of the TEPA-rGO/MXene nanocomposite prior to Nafion incorporation is shown in Figure 2A. SEM analysis reveals flake-like structures ranging from 3 to 10 μm in size, exhibiting a characteristic layered stacking behavior typical of two-dimensional materials. Elemental mapping via EDS (Figure 2B) shows the presence of key elements: carbon, oxygen, and nitrogen originating from TEPA-functionalized reduced graphene oxide (rGO-TEPA), and titanium, carbon, and oxygen originating from Ti_3_C_2_T_*x*_ MXene. To complement EDS analysis, XPS survey spectra were recorded (see the Supporting Information), revealing elemental compositions of carbon (58.7%), oxygen (19.61%), nitrogen (4.26%), and titanium (14.34%). Notably, fluorine (3.84%) was also detected, attributed to fluorinated terminal groups on the MXene surface.

**Figure 2:**
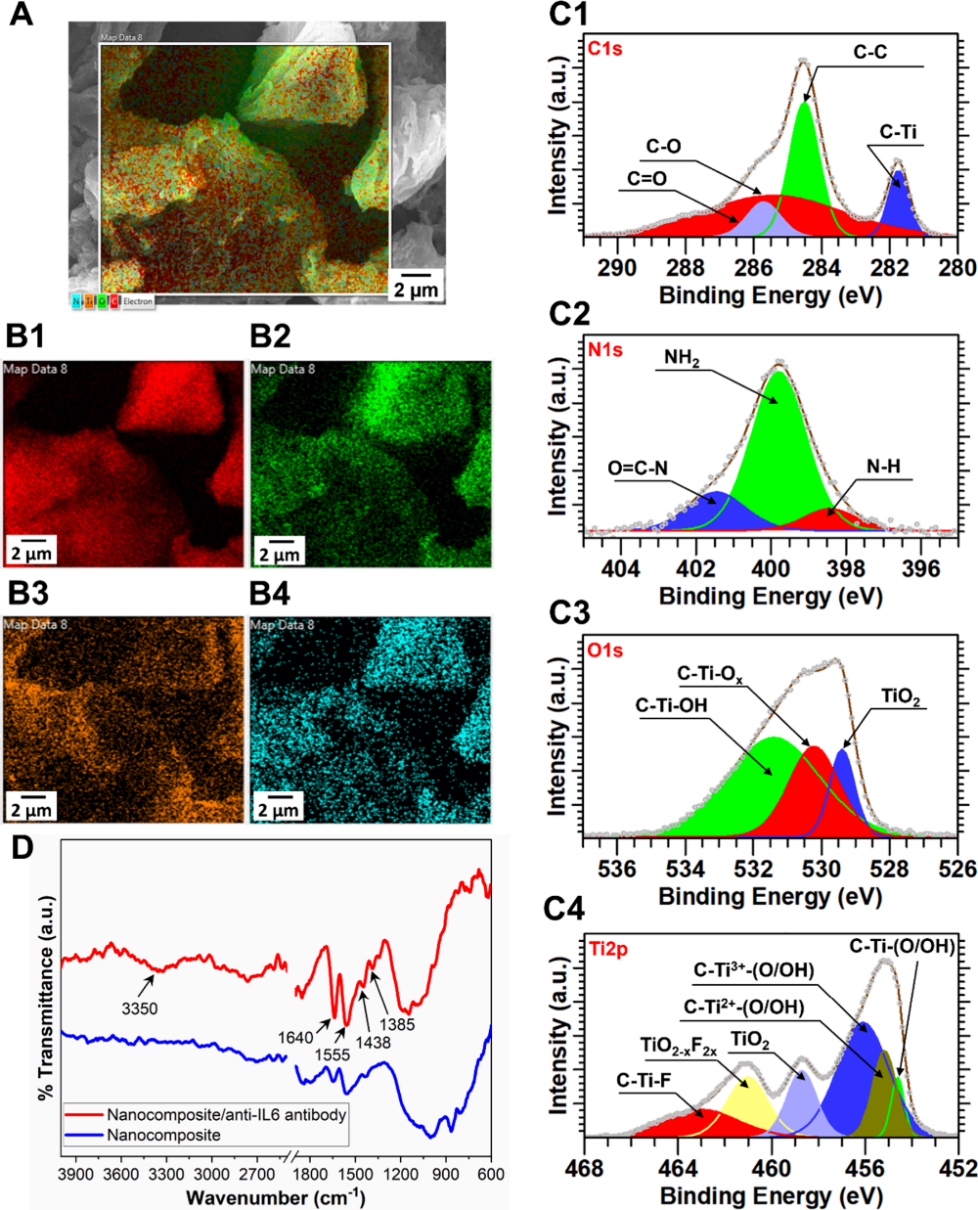
Morphological, elemental, and chemical characterization of the nanocomposite. (A) SEM image overlaid with elemental mapping. (B) EDX-based spatial distribution of key elements: (B1) carbon, (B2) oxygen, (B3) titanium, and (B4) nitrogen. (C) High-resolution deconvoluted XPS spectra illustrating the relative abundance of chemical linkages: (C1) C 1s, (C2) N 1s, (C3) O 1s, and (C4) Ti 2p. (D) FTIR spectra confirming covalent (amide) bond formation between the nanocomposite and the anti-IL-6 antibody via EDC–NHS coupling chemistry.

To gain further insight into the chemical states of the constituent elements, high-resolution XPS spectra for the C 1s, N 1s, O 1s, and Ti 2p regions were acquired and deconvoluted (Figure 2C). The C 1s spectrum was resolved into four components corresponding to C–C (284.51 eV), C–Ti (281.74 eV), C–O (285.43 eV), and C=O (285.71 eV).^31^ The C–Ti peak is characteristic of MXene, while the remaining peaks are associated with both rGO-TEPA and MXene. The N 1s spectrum was deconvoluted into three components assigned to NH (398.42 eV), NH_2_ (399.79 eV), and O=C–N (401.45 eV), all attributable to the TEPA-functionalized rGO.^32^ The dominant NH_2_ signal indicates that the majority (72.3%) of nitrogen species exist as primary amines, which are available for the covalent attachment of anti-IL-6 antibodies. The Ti 2p spectrum was fitted with six distinct peaks: C–Ti–(O/OH) (454.60 eV), C–Ti^2+^–(O/OH) (455.17 eV), C–Ti^3+^–(O/OH) (456.10 eV), TiO_2_ (458.71 eV), TiO_2–*x*_F_2*x*_ (461.02 eV), and C–Ti–F (461.03 eV).^33^ These chemical states are consistent with the complex surface chemistry of Ti_3_C_2_T_*x*_ MXene, including mixed oxidation states and fluorinated terminal groups.

Furthermore, FTIR spectroscopy was used to (Figure 2D) characterize the formation of EDC/NHS-based amide linkages between TEPA-rGO/MXene and the anti-IL-6 antibody, with distinctive FTIR peaks at 1743, 1640, 1510, 1440, and 1331 cm^–1^. The 1743 and 1510 cm^–1^ peaks confirm successful antibody immobilization through amide I (C=O stretching)^34^ and amide II (N–H bending and C–N stretching) linkages,^35^ respectively. The increased intensity at 1640 cm^–1^ is attributed to -NH_2_ groups in the Fc region of the antibody.^36^ The 1440 cm^–1^ peak corresponds to C–H bending vibrations in the lipid moieties, while multiple peaks below 1400 cm^–1^ represent the amide III band.^36^

Surface profile and wettability of the biosensor assembly were analyzed using AFM and static CA measurements (Figure 3). The bare WE exhibited a CA of 68° and an *R*_a_ of 7.5 nm. Coating the WE with TEPA-rGO/MXene/Nafion increased the CA to 108° and the *R*_a_ to 28.8 nm, attributed to Nafion’s inherent hydrophobicity, making it susceptible to nonspecific protein adsorption.^37^ After anti-IL-6 functionalization via EDC/NHS chemistry, the CA decreased to 78° and the *R*_a_ increased to 45 nm, indirectly confirming antibody immobilization. To enhance hydrophilicity and reduce protein fouling,^38^ a final blocking step with BSA was performed, significantly lowering the CA to 28° (*R*_a_ = 35 nm), thereby improving sensor specificity via biofouling mitigation.

**Figure 3:**
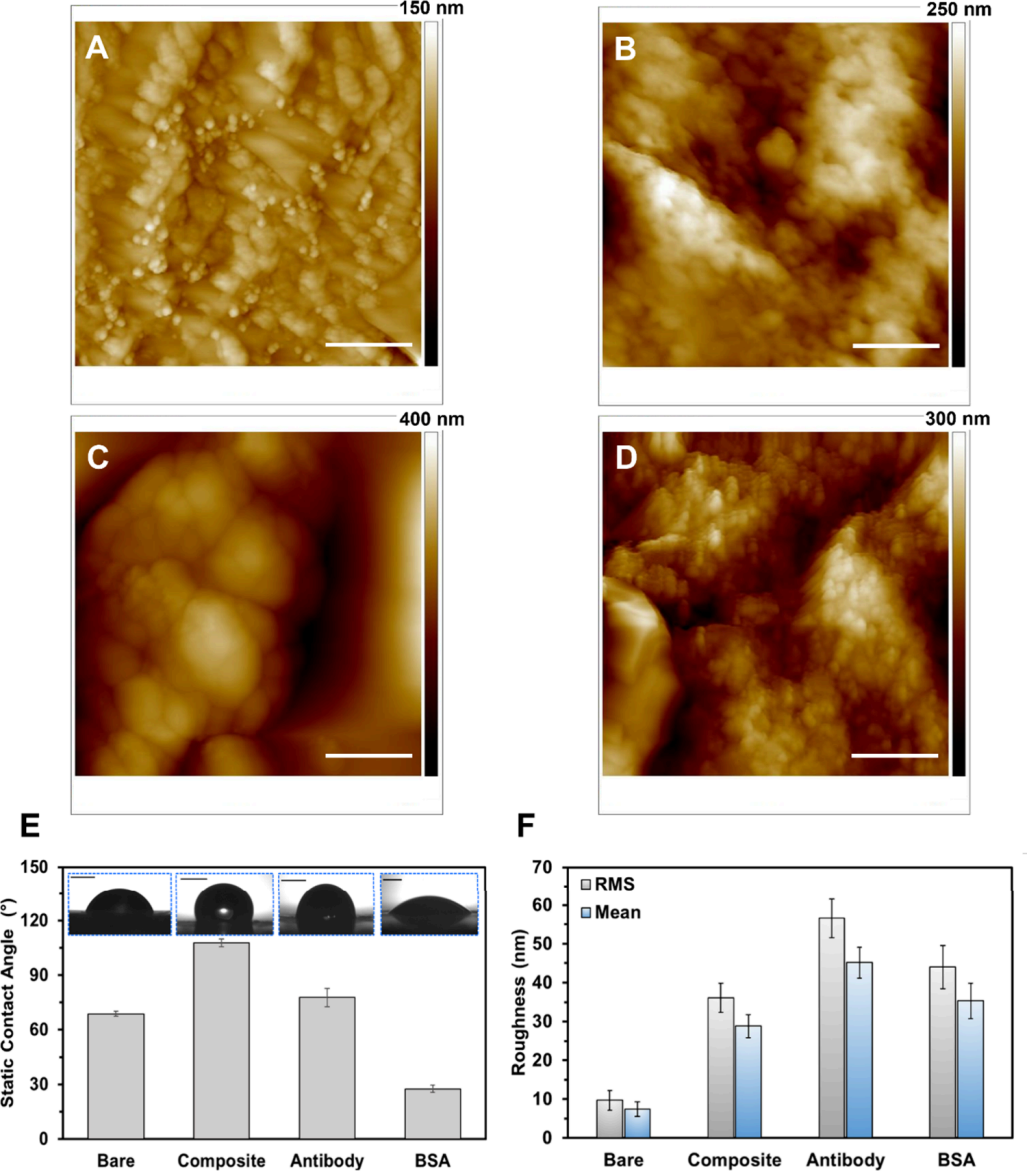
AFM and CA analysis of the stepwise assembly of the IL-6 sensor. (A–D) AFM micrographs of (A) bare WE, (B) nanocomposite-coated, (C) anti-IL-6 antibody-functionalized, and (D) BSA-blocked SPCEs. Scale bars represent 500 nm. (E) Static CA measurements of sequentially modified SPCEs, with insets displaying PBS droplet profiles on each surface (scale bars: 1 mm). (F) Quantitative analysis of surface roughness parameters—root-mean-square (RMS) and mean roughness—extracted from AFM images (A–D) over a 2 × 2 μm^2^ scan area. Error bars denote standard deviations based on measurements at three distinct regions on each electrode surface.

### 3.2 Optimal Nanocomposite Selection via Electrochemical Characterization

CV characterization was conducted across scan rates ranging from 25 to 200 mV/s to assess the electrochemical redox behavior of the IL-6 sensor fabrication protocol. The CV profiles were recorded within a potential window of −0.6 to +0.2 V using a 10 mM [Ru­(NH_3_)_6_]­Cl_3_ solution (Figure 4A). The binary nanocomposite TEPA-rGO/Nafion exhibited *I*_p_ ∼ 11.8 μA/mm^2^, which is 17% lower than that of bare WE, indicating a decrease in the interfacial conductivity and diffusion properties. Although the MXene/Nafion nanocomposite provided an *I*_p_ ∼ 21.6 μA/mm^2^ (i.e., 52% higher than bare WE), it resulted in higher Δ*E*_pp_ and background capacitive currents (*I*_b_), which is undesired. The *I*_p_/*I*_b_ ratio for the MXene/Nafion nanocomposite was evaluated as 1.6, which is 31% lower than that of bare WE, suggesting a loss in sensitivity. This was circumvented by the ternary nanocomposite TEPA-rGO/MXene/Nafion (*I*_p_ ∼ 29.7 μA/mm^2^) achieving an *I*_p_/*I*_b_ of 2.1, which is only 8.6% lower than that of the bare WE. Figure 4B confirms the linear relationship between *I*_p_ and *v* ^0.5^ attributed to a diffusion-controlled electron transport across for all of the nanocomposites.^29^ Furthermore, functionalizing the anti-IL-6 antibodies via EDC-NHS chemistry to the TEPA-rGO/MXene/Nafion nanocomposite drastically lowered the CV peak currents to *I*_p_ ∼ 13.6 μA/mm^2^ due to an increment in the charge transfer resistance and blocked redox probe diffusion after antibody immobilization. To determine the optimal concentration of constituents of the TEPA-rGO/MXene/Nafion nanocomposite for which *I*_p_/*I*_b_ is maximized, while Δ*E*_pp_ is lowered, systematic optimization was carried out. Figure 4C–E compare these metrics across varying concentrations of Nafion (0.25–1.5%), MXene (0.25–2 mg/mL), and TEPA-rGO (0.25–3 mg/mL) relative to the bare WE. These studies revealed that optimal *I*_p_/*I*_b_ and Δ*E*_pp_ were achieved at a specific concentration, beyond which increasing the material loading either reduced the interfacial conductivity or clogged the porous structure, ultimately hindering the thin-layer diffusion of [Ru­(NH_3_)_6_]^3+^.^29^ The final biosensor interface consisted of 0.5% Nafion, 0.5 mg/mL MXene, and 1 mg/mL TEPA-rGO suspended in DI water.

**Figure 4:**
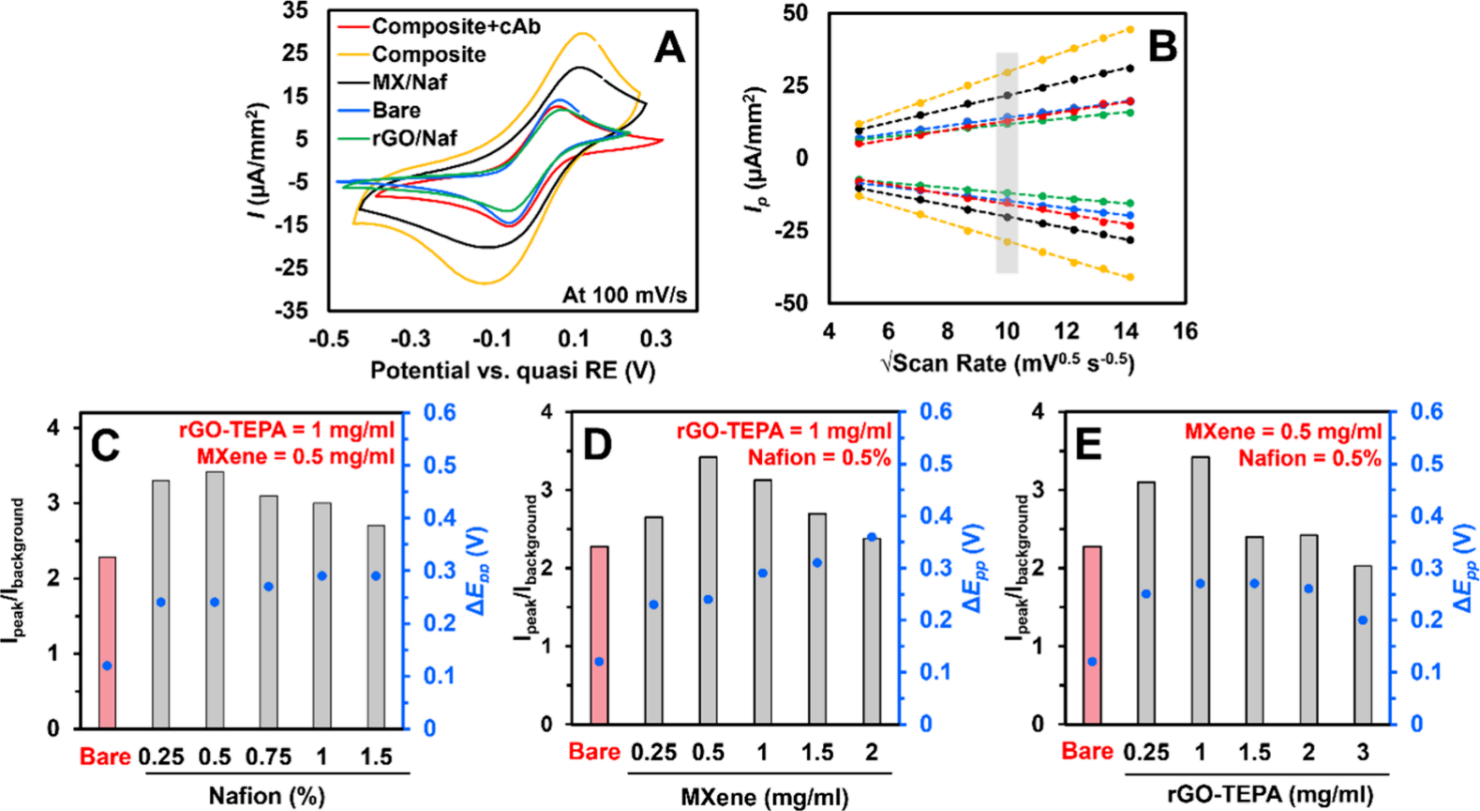
CV characterization of functionalized electrodes using 10 mM [Ru­(NH_3_)_6_]^3+^ in 100 mM KCl as the supporting electrolyte. (A) CV responses of SPCEs modified with various nanocomposites and anti-IL-6 antibodies, recorded at a scan rate of 100 mV/s. (B) Variation of oxidation and reduction peak currents (*I*_p_) as a function of the square root of the scan rate for different nanocomposites, indicating diffusion-controlled electron transfer. (C–E) Effects of varying concentrations of (C) TEPA-rGO, (D) MXene, and (E) Nafion on the background-normalized oxidation peak current (*I*_peak_/*I*_background_) and peak-to-peak potential separation (Δ*E*_pp_), providing insight into optimal sensor composition.

### 3.3 Redox Kinetics of the Electrochemical Sensor

The learned redox kinetic parameters throughout the stepwise fabrication of the EB are illustrated in Figure 5A,B. The bare WE has a diffusion coefficient of *D* ∼ 1 × 10^–5^ cm^2^/s and a rate constant of *k*_0_ ∼ 4.5 × 10^–3^ cm/s, which is in good agreement with planar electrodes as per the electrochemistry literature.^39^ Modifying the WE with the TEPA-rGO/MXene/Nafion nanocomposite enhances the apparent diffusion coefficient to *D* ∼ 6 × 10^–5^ cm^2^/s, which is six times greater than that of bare WE. This shift indicates a transition from semi-infinite planar diffusion to thin-layer diffusion.^29^ However, this enhancement comes at an expense of a 2-fold reduction in kinetics, yielding a *k*_0_ ∼ 2.1 × 10^–3^ cm/s. This implies that while the porous nanocomposites facilitate the increased diffusion of [Ru­(NH_3_)_6_]^3+^, they also increase the irreversibility of the CV reaction, ultimately requiring an overpotential for the Faradaic peak to occur. Upon covalently functionalizing the nanocomposite with the anti-IL-6 antibody, the diffusion characteristic reverts to similar levels to the bare WE (*D* ∼ 1.2 × 10^–5^ cm^2^/s), while the kinetics improve significantly (*k*_0_ ∼ 6.5 × 10^–3^), lowering the irreversibility for the redox reaction [*Ru*(*NH*_3_)_6_]^3+^ ⇌ [*Ru*(*NH*_3_)_6_]^3+^. Furthermore, the surface concentration of cAb was evaluated using the Brown–Anson model,^40^ which has the form $\gamma =\frac{4RTI_{\mathrm{ox}}A_{g}}{F^{2}v}\times \frac{1}{\mathrm{ECSA}}$ . Utilizing the electrochemical parameters of the cAb-functionalized electrode *I*_ox_ = 12.6 μA/mm^2^ for *v* = 0.1 V/s (Figure 4B) and ECSA = 0.1125 cm^2^ (Figure 5B), the surface concentration of the anti-IL-6 antibody is estimated as 25.1 nmol/cm^2^.

**Figure 5:**
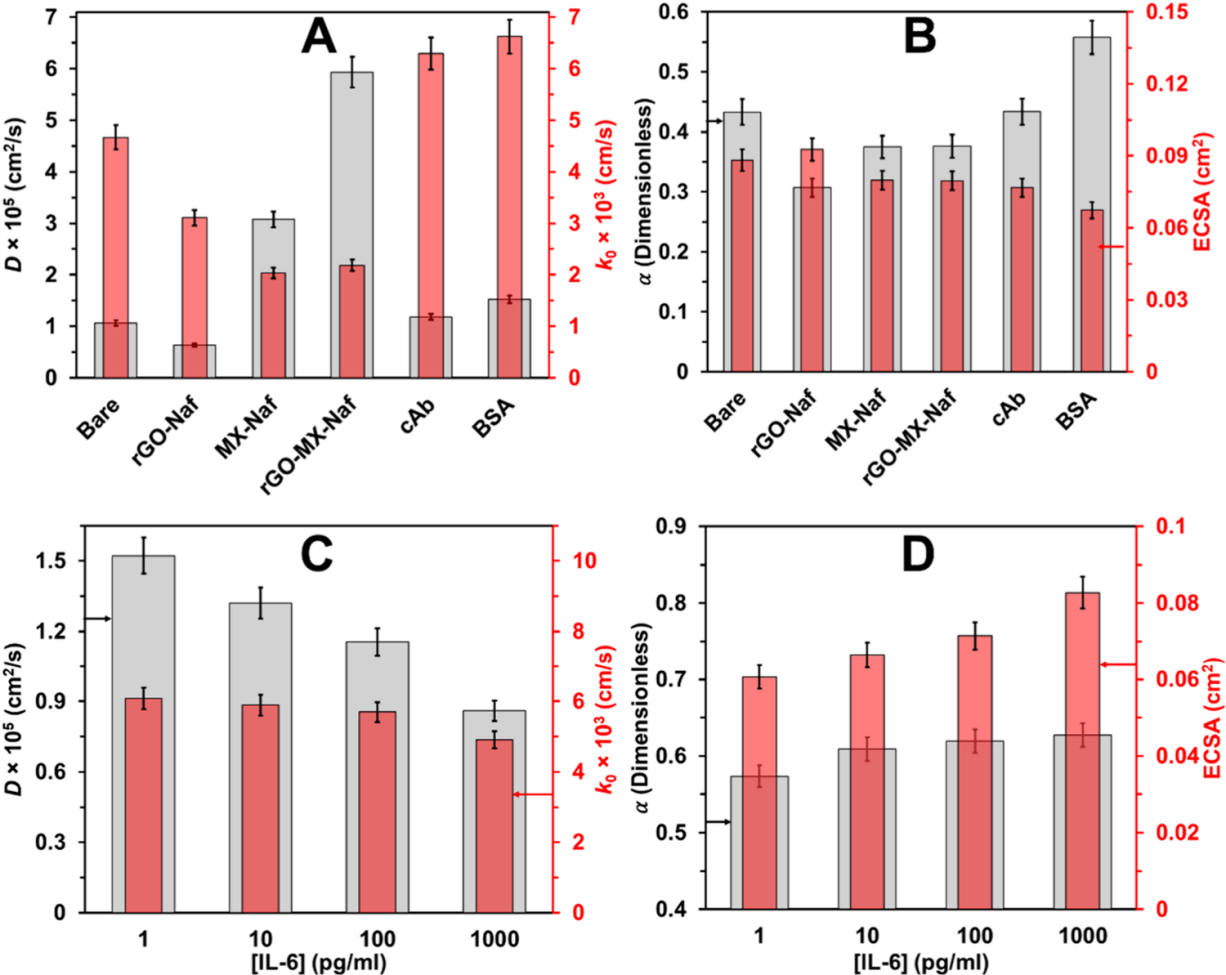
Electrochemical parameters extracted using a GA-based diffusion model. (A) Effect of electrode surface modification on the diffusion coefficient (*D*) and standard heterogeneous electron transfer rate constant (*k*_0_). (B) Influence of functionalization on the charge transfer coefficient (α) and the *ECSA*. (C,D) Changes in electrochemical parameters resulting from IL-6 antigen–antibody interactions, highlighting their impact on sensor performance. The error bars in all figures represent a 5% variation, derived from five independent runs of the stochastic GA framework.

Another critical aspect is the use of the inverse model to quantify changes in the electrochemical transport parameters as the antigen–antibody complex formation varies (Figure 5C,D). Increasing the Il-6 concentration from 1 to 1000 pg/mL leads to a monotonic decrease in both *D* and *k*_0_ values, indicating that the increased antigen–antibody complex hinders the diffusion and kinetics across the biosensing interface. The increment in ECSA (Figure 5D) can be attributed to the partial neutralization of surface charge after IL-6 binding, implying reduction in impedance.^41^

The physical parameters inferred using the GA-coupled FVM-based thin-layer diffusion model successfully capture previously unknown information in a physically consistent and interpretable manner. This hybrid framework is well aligned with contemporary inverse problem-solving strategies, preserving mechanistic fidelity through embedded diffusion equations, while leveraging the optimization capabilities of evolutionary algorithms. In contrast to conventional machine learning models (e.g., neural networks or regression techniques), which often lack inherent physical constraints and may produce nonphysical parameter estimates, our approach ensures that the inferred parameters remain both mechanistically meaningful and experimentally reliable.

### 3.4 Performance of the Label-Free IL-6 Sensor

Human serum samples spiked with varying IL-6 concentrations were incubated with the sensors followed by CV measurements using [Ru­(NH_3_)_6_]^3+^ as the redox probe. An increase in the reduction peak current around −0.3 V was consistently observed with higher IL-6 concentrations (Figure 6A), indicating a decrease in charge transfer resistance and enhanced electron transfer during antigen–antibody complex formation. This effect is attributed to the anti-IL-6 functionalized TEPA-rGO/MXene/Nafion nanocomposite layer, which initially acts as a precharged capacitor, hindering electron transfer from the redox probe. Upon IL-6 binding, a redistribution of surface charge occurs, partially neutralizing the capacitive charge and reducing impedance, leading to an increase in peak current.^41^ The three-orders-of-magnitude increase in IL-6 binding led to a 30% expansion in the *ECSA*, enhancing the electron tunneling efficiency (Figure 5D).

**Figure 6:**
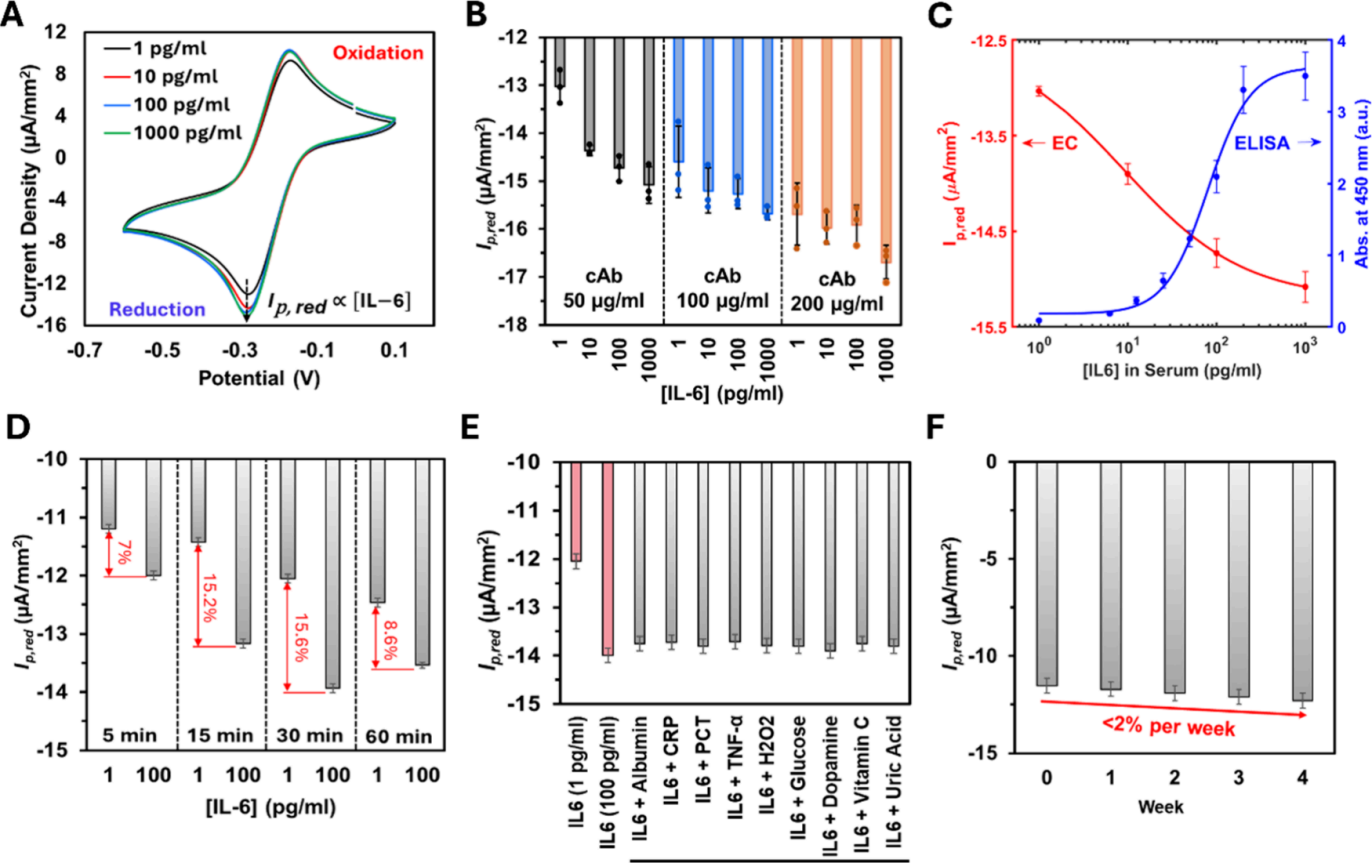
Sensitivity, specificity, and stability assessment of the IL-6 electrochemical sensor. (A) CV responses of the sensor exposed to varying concentrations of IL-6 spiked into human serum. (B) Optimization of cAb concentration, with bar graphs representing the average reduction peak current (*I*_p_,_red_). (C) Calibration curves generated using 4PL regression for the electrochemical sensor (red; utilizing 50 μg/mL cAb) and a commercial ELISA kit (blue), both tested with IL-6-spiked serum. The *x*-axis is in logarithmic scale. (D) Optimization of sample incubation time to maximize current response. (E) Specificity analysis showing the target IL-6 detection signal (red bar) and responses to nontarget serum interferents (gray bars), indicating minimal cross-reactivity. (F) Long-term stability of the IL-6 sensor evaluated over a one-month period. Error bars in all panels represent the standard deviation of the mean for *n* = 3 replicates.

A parametric study identified 50 μg/mL as the optimal concentration for the anti-IL-6 cAb, balancing the IL-6 binding efficiency with minimal charge hindrance (Figure 6B). Higher cAb concentrations (e.g., 100 and 200 μg/mL) resulted in steric hindrance driven by surface crowding and reduced sensitivity at lower IL-6 levels, which are of clinical interest. It is important to note that the relative standard deviations between replicates for these experiments were less than 5%.

A four-parameter logistic (4PL) calibration curve was fitted^42,43^ to the current vs concentration data for the electrochemical sensor using a 50 μg/mL cAb concentration (Figure 6C), based on the data shown in Figure 6B. In parallel, a commercial IL-6 ELISA kit was employed to quantify IL-6 levels in the same serum samples, with 4PL regression applied to generate the corresponding calibration curve. The 4PL model is defined as

$$y=d+\frac{a-d}{1+{\left(\frac{x}{c}\right)}^{b}}$$ 7

where *x* is the IL-6 concentration (pg/mL) and *y* is the sensor response, measured as the reduction peak current (μA/mm^2^) for the electrochemical sensor and absorbance (a.u.) for ELISA. The parameters *a*, *b*, *c*, and *d* represent the asymptotic minimum, Hill slope, inflection point (EC_50_), and asymptotic maximum, respectively, and were derived via nonlinear regression (*R* ^2^ ∼ 0.97).

For the electrochemical sensor, the fitted 4PL parameters were *a* = −15.2, *b* = −0.64, *c* = 8.92, and *d* = −12.5. For ELISA, the corresponding values were *a* = 0.183, *b* = 1.83, *c* = 79.4, and *d* = 3.63. The limit of detection (LOD) for each platform was calculated according to the IUPAC definition, as 3.3 times the standard deviation of the lowest concentration group exhibiting a statistically significant signal (*p* < 0.05). The LOD for the electrochemical sensor was determined to be 2.1 pg/mL, while that of the ELISA kit was 5 pg/mL. The linear detection range was 3–200 pg/mL for the electrochemical sensor and 5–100 pg/mL for the ELISA. When plotted on a logarithmic scale, both methods demonstrated a dynamic range extending up to 1000 pg/mL, with the electrochemical sensor showing broader coverage starting from 3 pg/mL. These results highlight the EB’s competitive sensitivity and dynamic range when compared to the commercial ELISA, with a much lower turnaround time.

Selectivity was tested by spiking serum samples with 100 pg/mL IL-6 and potential interferents, 10 mg/mL BSA, sepsis-related proteins (1000 pg/mL C-reactive protein, 100 pg/mL procalcitonin, and 100 pg/mL tumor necrosis factor-α), and physiologically relevant concentrations of common electroactive and small-molecule interferents (e.g., 70 mg/dL glucose, 1 μM hydrogen peroxide, 50 μM vitamin C, 50 pg/mL dopamine, and 5 mg/dL uric acid).^9^ The maximum interference observed was only 14% of the signal detected from specific IL-6 binding, demonstrating the biosensor’s high selectivity (Figure 6E). The signal-to-noise ratio was evaluated using the following expression:

$$\mathrm{SNR}=\frac{I_{p,\mathrm{red}}^{\mathrm{target}}-I_{p,\mathrm{red}}^{\mathrm{background}}}{I_{p,\mathrm{red}}^{\mathrm{int}\mathrm{erf}\mathrm{errent}}-I_{p,\mathrm{red}}^{\mathrm{background}}}$$ 8

where *I*_*p*,red_^target^ is the current response for 100 pg/mL IL-6, *I*_*p*,red_^background^ is the current response for 1 pg/mL IL-6, and *I*_*p*,red_^interferrent^ is the current response from 100 pg/mL spike mixed with any known concentration of interferents from the above-mentioned list. This results in an impressive mean signal-to-noise ratio of 6.9, which is well above the widely accepted minimum SNR threshold of 3.^9^

Stability assessment of the developed sensor over a four-week period at 4 °C revealed minimal variation in peak current, with a maximum decline of approximately 2% per week, demonstrating excellent long-term stability (Figure 6F). The label-free IL-6 sensor exhibited high selectivity, stability, and a rapid response time with sensitivity comparable to previously reported approaches (see Supporting Information), underscoring its promise for POC diagnostic applications. To further improve the shelf life and enable storage at ambient conditions, ongoing and future work is focused on the development of antibody-free IL-6 sensors employing molecularly imprinted polymers as synthetic recognition elements.

## 4 Conclusions

We developed a label-free IL-6 sensor exhibiting clinically relevant sensitivity, along with enhanced specificity and stability, by incorporating a TEPA-rGO/Ti_3_C_2_T_*x*_-based nanocomposite within a Nafion matrix. The sensor demonstrated rapid and reliable detection of IL-6 in serum samples, achieving a detection limit as low as 2.1 pg/mL and maintaining consistent performance across a wide dynamic range of 3–1000 pg/mL. The integration of a thin-layer diffusion model with a GA optimization framework not only enabled precise parameter estimation but also provided deeper insights into the reaction-diffusion mechanisms at the biosensor interface. Comprehensive characterizations confirmed the robust design and functionality of the nanocomposite, including its morphology, chemical structure, antibody attachment, and wetting properties, which significantly reduced nonspecific protein adsorption and enhanced sensor selectivity. These results underline the potential of this platform to serve as a reliable and efficient POC diagnostic tool for IL-6 and potentially other biomarkers relevant to inflammatory diseases. The integration of computational modeling with advanced nanomaterial design represents a powerful approach for enhancing biosensor performance. Future work could explore multiplexing capabilities and further miniaturization to enable the simultaneous detection of multiple biomarkers for comprehensive disease profiling.

## Supplementary Material



## Notes

Appendix 1, detailed numerical methodology for discretization of the governing differential equations; Appendix 2, grid independence study conducted for the thin-layer diffusion model; Appendix 3, comparison of the sensor developed in this work to previous electrochemical IL-6 sensors; Appendix 4, protocol for the commercial sandwich ELISA to quantify IL-6 protein concentrations; and Appendix 5, XPS survey spectra of the nanocomposite (PDF)
