# Supplementary material for: Label-Free Electrochemical Interleukin‑6 Sensor Exploiting rGO-Ti3C2T x MXene Nanocomposites
Source: ACS Appl Mater Interfaces. 2025 Jul 24;17(31):44112–22. doi: 10.1021/acsami.5c06701 (PMC12332829; doi:10.1021/acsami.5c06701)
Supplement: Supplementary file 1 [file am5c06701_si_001.pdf]

Supporting Information

Label-free Electrochemical Interleukin-6 Sensor Exploiting rGO-$Ti_3C_2T_x$ MXene Nanocomposite

Rohit Gupta [1, 2, #], Ashish Kalkal [1, 2, #], Priya Mandal [1, 2], Diptiranjan Paital [3], David Brealey [4,5], Manish K. Tiwari [1, 2, 6 *]

[1] Nanoengineered Systems Laboratory, UCL Mechanical Engineering, University College London, London, WC1E 7JE, UK

[2] UCL Hawkes Institute, University College London, London, W1W 7TS, UK

[3] Department of Physics, King's College London, London, WC2R 2LS, UK

[4] Division of Critical Care, University College London Hospitals, London, NW1 2BU, UK

[5] NIHR University College London Hospitals Biomedical Research Centre, London, NW1 2BU, UK

[6] Manufacturing Futures Laboratory, University College London, London, E20 2AE, UK

[#] The authors contribute equally

Email: m.tiwari@ucl.ac.uk, Phone: +44 20 3108 1056 (Manish K. Tiwari)

**Topic**	**Page No.**
Appendix 1: Discretization of Governing Equations	3
Appendix 2: Grid Independence Study	7
Appendix 3: Comparative Assessment with Previous Label-free IL-6 sensors	8
Appendix 4: Sandwich ELISA Protocol	10
Appendix 5: XPS survey spectra for the nanocomposite	11

Appendix 1: Discretization of Governing Equations

Fick's 2nd law (Eq. 1), Butler-Volmer equation (Eq. 2), far-field boundary condition (Eq. 3), and initial conditions (Eq. 4) stated in the main paper are discretised within the spatiotemporal domain using finite volume method (FVM) with *N* equally spaced grid points (see Figure S1) and using implicit time marching scheme for unconditional numerical stability.

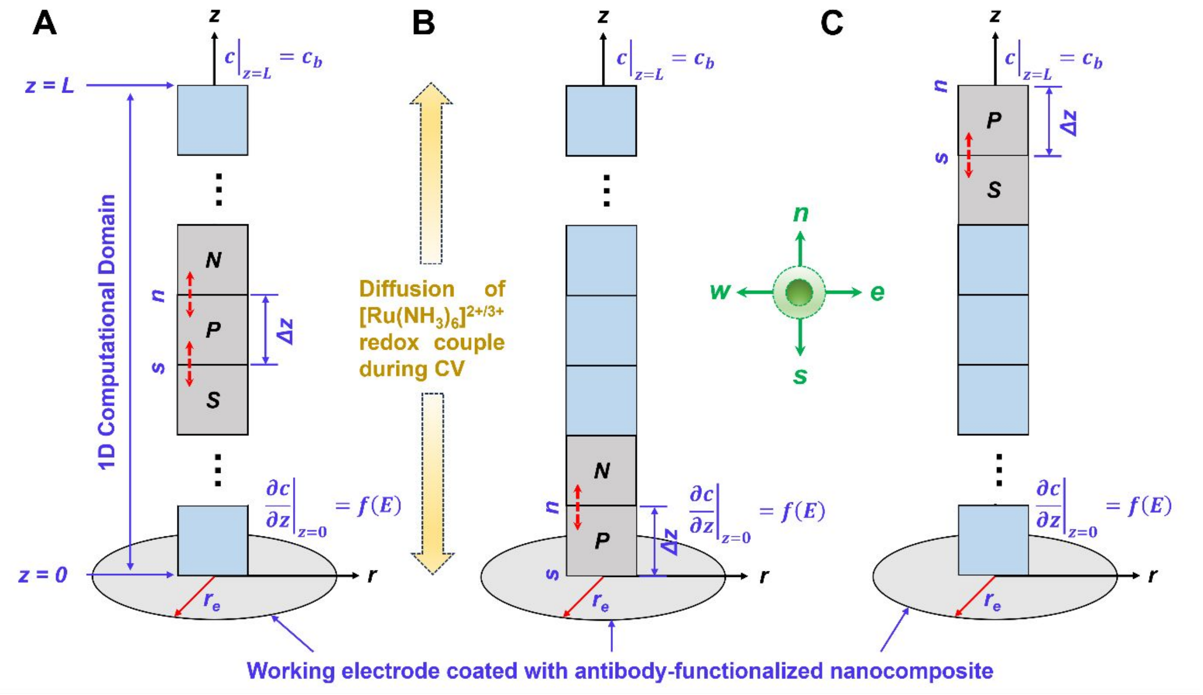

Figure S1. Representative schematic illustrating spatial discretization using finite volume notation for (A) interior nodes, (B) electrode-adjacent boundary nodes, and (C) far-field boundary nodes. The control volume of interest is labelled as *P*, with its neighbouring top and bottom control volumes designated as *N* and *S*, respectively. The interface between the control volumes *N* and *P* is marked as *n*, and between *P* and *S* as *s*, where diffusion of redox species occurs. The total number of control volumes within the domain is determined through a grid independence study. Here, r_e = 2 cm represents the radius of the circular working electrode in the screen-printed carbon electrode (SPCE) chip. For simplicity, the antibody-functionalized nanocomposite layer coated on the working electrode is not depicted.

Integrating Fick's 2nd law over span of control volume Δz and timestep Δt gives,

$$\int_t^{t+\Delta t}\int_s^n \frac{\partial c(z,t)}{\partial t}\,dzdt = \int_t^{t+\Delta t}\int_s^n D\frac{\partial^2 c(z,t)}{\partial z^2}\,dzdt \tag{A1}$$

$$\Rightarrow \left[c_P^{t+\Delta t} - c_P^t\right]\Delta z = \int_t^{t+\Delta t} \left[D_n \frac{\partial c}{\partial z}\bigg|_n - D_s \frac{\partial c}{\partial z}\bigg|_s \right] dt \tag{A2}$$

The quantities with subscript *P* are evaluated at the central location of the CV, whereas those with subscripts *n* and *s* are determined at the faces of the CV. For implicit time scheme, isotropic diffusion coefficient (i.e., $D_n = D_s = D$), and piecewise linear profile assumption for $\partial c/\partial z$, Eq. (A2) is rewritten as,

$$\Rightarrow \left[c_P^{t+\Delta t} - c_P^t\right]\Delta z = D\left[\frac{c_N^{t+\Delta t} - c_P^{t+\Delta t}}{\Delta z} - \frac{c_P^{t+\Delta t} - c_S^{t+\Delta t}}{\Delta z}\right]\Delta t \tag{A3}$$

Furthermore, Eq. (A3) can be written in simplified notations as follows.

$$\Rightarrow a_P^{t+\Delta t} c_P^{t+\Delta t} = a_N^{t+\Delta t} c_N^{t+\Delta t} + a_S^{t+\Delta t} c_S^{t+\Delta t} + a_P^t c_P^t + b \tag{A4}$$

where $a_N^{t+\Delta t} = a_S^{t+\Delta t} = \frac{D}{\Delta z}$, $a_P^{t+\Delta t} = \frac{\Delta z}{\Delta t} + \frac{2D}{\Delta z}$, $a_P^t = \frac{\Delta z}{\Delta t}$, and $b = 0$. Considering that the spatial domain is discretized in *N* equally spaced grid points, these coefficients are valid for the interior nodes (i.e., *i* = 2 to *N*-1). To obtain these coefficients for *i* = 1and *i* = *N,* the electrode-adjacent Butler-Volmer (B-V) and far-field BCs are required, respectively.

The B-V equation describes the redox reactions dependent on potential sweep during CV measurements at the electrode-adjacent boundary (*z* = 0). By substituting the B-V equation into Eq. (A2), the FVM formulation for the electrode boundary is derived, as illustrated in Figure S1B.

$$\left[c_P^{t+\Delta t} - c_P^t\right]\frac{\Delta z}{\Delta t} = D\frac{c_N^{t+\Delta t} - c_P^{t+\Delta t}}{\Delta z} - k_0\left[c_P^{t+\Delta t}\exp\left\{-\frac{\alpha F\left(E - E_f^0\right)}{RT}\right\} - \left(c_b - c_P^{t+\Delta t}\right)\exp\left\{\frac{(1-\alpha)F\left(E - E_f^0\right)}{RT}\right\}\right] \tag{A5}$$

Rearranging Eq. (A5) to obtain the standard FVM form (Eq. (A4)), the coefficients are $a_N^{t+\Delta t} = D/\Delta z$, $a_S^{t+\Delta t} = 0$, $a_P^{t+\Delta t} = \frac{\Delta z}{\Delta t} + \frac{D}{\Delta z} + k_0\left[\exp\left\{-\frac{\alpha F(E-E_f^0)}{RT}\right\} + \exp\left\{\frac{(1-\alpha)F(E-E_f^0)}{RT}\right\}\right]$, $a_P^t = \Delta z/\Delta t$, and $b = k_0 c_b \exp\left\{\frac{(1-\alpha)F(E-E_f^0)}{RT}\right\}$.

As the far-field boundary condition (*z* = *L*) remains unaffected by the CV potential sweep at the electrode, a constant concentration of redox species $c|_{z=L,t>0} = c_b$ is substituted into Eq. (A2) to calculate the flux through face *n*.

$$\left[c_P^{t+\Delta t} - c_P^t\right]\frac{\Delta z}{\Delta t} = D\left[\frac{c_b - c_P^{t+\Delta t}}{\Delta z/2} - \frac{c_P^{t+\Delta t} - c_S^{t+\Delta t}}{\Delta z}\right] \tag{A6}$$

Referring to Figure S1C, the far-field boundary condition $c|_{z=L,t>0} = c_b$ is imposed at a distance $\Delta z/2$ from the node *P*, for which the $\left.\frac{\partial c}{\partial z}\right|_n$ is evaluated over half of the CV, resulting to $\frac{c_b - c_P^{t+\Delta t}}{\Delta z/2}$. The coefficients for far-field boundary are $a_N^{t+\Delta t} = 0$, $a_S^{t+\Delta t} = \frac{D}{\Delta z}$, $a_P^{t+\Delta t} = \frac{\Delta z}{\Delta t} + \frac{3D}{\Delta z}$, $a_P^t = \frac{\Delta z}{\Delta t}$, and $b = \frac{2Dc_b}{\Delta z}$. The above-derived coefficients for the generic FVM equation (Eq. (A4)) are summarized in Table S1.

Table S1. Coefficients for Eq. (A4) across the entire computational domain.

Node	$a_N^{t+\Delta t}$	$a_S^{t+\Delta t}$	$a_P^{t+\Delta t}$	a_P^t	b
Electrode Boundary (z = 0)	$\frac{D}{\Delta z}$	0	$\frac{\Delta z}{\Delta t}+\frac{D}{\Delta z} + k_0\left[\exp\left\{-\frac{\alpha F\left(E-E_f^0\right)}{RT}\right\} + \exp\left\{\frac{(1-\alpha)F\left(E-E_f^0\right)}{RT}\right\}\right]$	$\frac{\Delta z}{\Delta t}$	$k_0 c_b \exp\left\{\frac{(1-\alpha)F\left(E-E_f^0\right)}{RT}\right\}$
Interior Nodes (0 < z < L)	$\frac{D}{\Delta z}$	$\frac{D}{\Delta z}$	$\frac{\Delta z}{\Delta t}+\frac{2D}{\Delta z}$	$\frac{\Delta z}{\Delta t}$	0
Bulk Boundary (z = *L*)	0	$\frac{D}{\Delta z}$	$\frac{\Delta z}{\Delta t}+\frac{3D}{\Delta z}$	$\frac{\Delta z}{\Delta t}$	$\frac{2Dc_b}{\Delta z}$

Appendix 2: Grid Independence Study

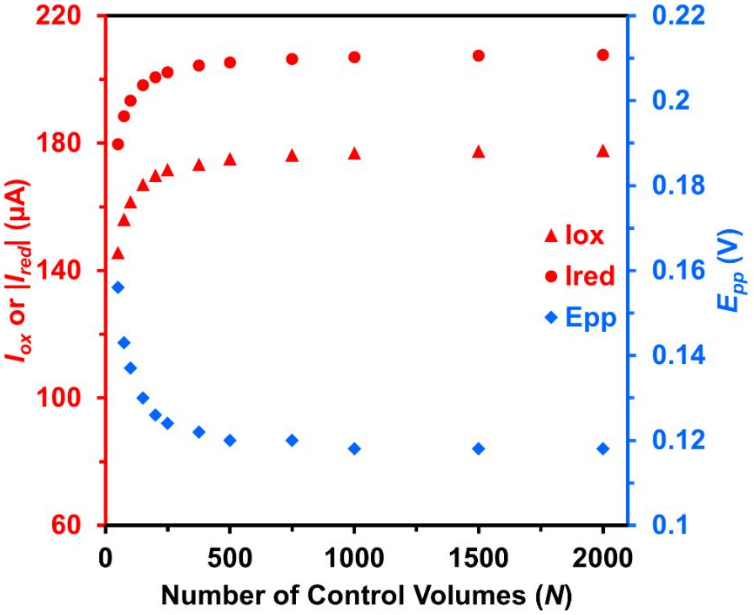

Figure S2. The grid independence study illustrates the variations in peak-to-peak potential separation (E_{pp}) represented by blue diamonds, oxidation peak current (I_{ox}) represented by red triangles, and the absolute value of reduction peak current ($|I_{red}|$) represented by red circles, as a function of the number of control volumes in the FVM discretization. This validation was conducted for the CV simulation of a bare electrode, with a specified simulation domain length (L) of 0.0734 cm. Based on this analysis, a control volume number of N = 500 was selected for subsequent coupling with the GA optimization solver, as it provided an accurate representation of the underlying physics while ensuring a code execution time of less than 5 seconds.

Appendix 3: Comparative Assessment with Previous Label-free IL-6 sensors

Table S2. Literature review comparing the Il-6 sensor developed in this work with previous efforts in terms of nanomaterial interface, analytical performance, and sample matrix.

Sensor Material	Measurement Technique	LoD	Range (pg/ml)	Specificity Testing Analyte	Stability (weeks)	Sample incubation time and temperature	Sample Matrix	Ref.
Au Microneedle	DPV, EIS	40 pg/ml	1-80 pg/ml	BSA, Enterotoxin A	NR	15 min, 25°C	Serum (Spiked)	1
AuNPs/ pATP/pABA/GCE	EIS	1.66 pg/ml	5-100,000 pg/ml	CEA, MUC1, MUC4, MUC16	NR	60 min, 25°C	Serum (Clinical)	2
Au/PEDOT:PSS	V_{GS} *vs.* I_{SD} characteristics	24 pM	0.1-10 nM	TNF-α	NR	15 min, 25°C	Tween20 Buffer	3
Au/ZnO/DSP	EIS	0.1 pg/ml	0.01-10,000 pg/ml	IL-8, IL-10, IP-10, TRAIL	5	5 min, 25°C	Plasma (Clinical)	4
Au/MPA	EIS, DPV	11.8 pg/ml	10-10,000 pg/ml	TNF-α, IL-10	NR	30 min, 27°C	Serum (spiked), CSF	5
ITO/PPy-Epoxy	EIS, CV	6 fg/ml	0.02-16 pg/ml	BSA	2	30 min, 25°C	Serum (Spiked)	6
ITO/PPy-NHS	EIS, CV	10.2 fg/m	0.03-22.5 pg/ml	Not tested	2	45 min, 25°C	Serum (Spiked)	7
Si-SiO2/SWCNT/AuNP	EIS	0.01 fg/m	0.01-100 fg/ml	Glucose, Cysteine, Epinephrine	4	30 min, 25°C	Serum (Spiked)	8
CCY/α-Fe_2O_3 NR	CV	0.26 pg/ml	1-100,000 pg/ml	IgG, PSA, HSA, IL-8, IL-10,	3	Not reported	PBS	9
CF/AuNF/MPA	CV, DPV	1 pg/ml	1-1000,000 pg/ml	CEA, IL-8, IL-10, PCT, HAS,	1.5	5 min, 25°C	Serum (Spiked)	10

				PSA, IgG, Cytc, AFP				
CNT/Graphene Fiber	SWV	0.38 pg/ml	0.001-10,000 pg/ml	BSA, HSA, DCD, IL-8	1	30 min, 25°C	Sweat (Clinical)	[11]
SPCE/PPy/AuNP	EIS	0.33 pg/ml	1-15,000,000 pg/ml	CEA, BSA, MUC1, MUC4, MUC16,	1.5	30 min, 37°C	Serum (Spiked)	[12]
SPCE/Biochar	SWV	8.8 pg/ml	26-140 pg/ml	IL-1B, IL-2, IL-5, IL-12, TNF-α	2.5	15 min, 25°C	Serum (spiked), blood (spiked)	[13]
SPCE/TEPA-rGO/MXene/Nafion (Current work)	CV	2.1 pg/ml	3-1000 pg/ml	BSA, PCT, CRP, TNF-α, GFAP, IP-9, IP-10	4	15 min, 25°C	Serum (spiked)	This work

Appendix 4: Sandwich ELISA Protocol for IL6 Detection in Serum Samples

The sandwich assay for IL-6 detection in serum samples was conducted using NUNC MaxiSorp flat-bottom transparent 96-well plates. The following protocol adheres to the manufacturer's instructions for the CHC1263 Human IL-6 CytoSet ELISA kit (Invitrogen). Initially, the anti-human IL-6 capture antibody stock (1 mg/ml) was diluted to a concentration of 1 µg/ml in 1X coating buffer (prepared from 10X PBS, Invitrogen). A volume of 100 µl of the diluted capture antibody was added to each well, after which the plate was covered with parafilm to minimize evaporation and incubated overnight at 4°C. Excess solution was removed, and the wells were washed three times with 1X wash buffer (prepared from 20X wash buffer containing 0.05% Tween 20 and 1X PBS). To block non-specific binding sites, 200 µl of 1X assay buffer (prepared from 5X ELISA/ELISPOT buffer) was added to each well, followed by a 2-hour incubation at room temperature. The excess liquid was then removed, and the wells were washed three times with 1X wash buffer. Next, eight different concentrations (ranging 1 to 1000 pg/ml) of IL-6 protein-spiked human serum samples were added to the wells in triplicate and incubated for 2 hours at room temperature. After removing excess liquid and washing with 1X wash buffer, 100 µl of 0.16 µg/ml biotinylated anti-human IL-6 detection antibody (prepared from a 200 µg/ml stock) in 1X assay buffer was added to each well. Following a 1-hour incubation, the wells were washed four times after removing the excess liquid. A 1/2500 dilution of streptavidin-conjugated HRP, supplied with the kit, was prepared in 1X assay buffer, and 100 µl was added to each well. After a 30-minute incubation, the wells were washed up to five times with 1X wash buffer following removal of the excess liquid. Subsequently, 100 µl of TMB (Invitrogen) was added to each well and incubated for 15 minutes in the dark. The enzymatic reaction was terminated by adding 100 µl of stop solution (1M H_2SO_4) to each well, stabilizing the yellow color of the solution. Absorbance values were measured using a Tecan Infinite 200Pro microplate reader at wavelengths of 450 and 570 nm. For baseline correction, the absorbance at 570 nm was subtracted from that at 450 nm.

Appendix 5: XPS survey spectra of the nanocomposite

The full XPS spectrum of the nanocomposite, presented in Figure S3, confirms the presence of key elements including C 1s (285 eV, 58.7%), N 1s (400 eV, 4.26%), Ti 2p (456 eV, 14.34%), O 1s (530 eV, 19.61%), and F 1s (685 eV, 3.84%). Carbon and oxygen are contributed by both rGO-TEPA and $Ti_3C_2T_x$ MXene, whereas titanium and fluorine are exclusively derived from the MXene component. The nitrogen signal originates from the functional groups of rGO-TEPA, which is employed to functionalize the anti-IL-6 antibodies.

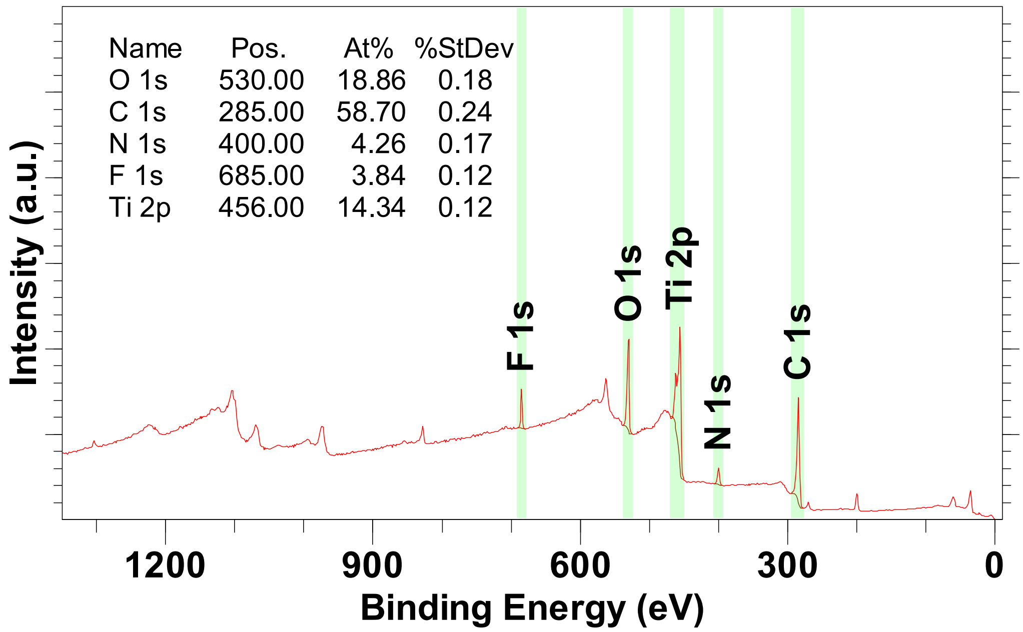

Figure S3. X-ray photoelectron full spectra of the nanocomposite marked with constituent peaks C 1s, N 1s, Ti 2p, O 1s, and F 1s.

References

1. Russell, C.; Ward, A. C.; Vezza, V.; Hoskisson, P.; Alcorn, D.; Steenson, D. P.; Corrigan, D. K., Development of a needle shaped microelectrode for electrochemical detection of the sepsis biomarker interleukin-6 (IL-6) in real time. *Biosensors and Bioelectronics* **2019,** *126*, 806-814.
2. Tertis, M.; Leva, P. I.; Bogdan, D.; Suciu, M.; Graur, F.; Cristea, C., Impedimetric aptasensor for the label-free and selective detection of Interleukin-6 for colorectal cancer screening. *Biosensors and Bioelectronics* **2019,** *137*, 123-132.
3. Diacci, C.; Burtscher, B.; Berto, M.; Ruoko, T.-P.; Lienemann, S.; Greco, P.; Berggren, M.; Borsari, M.; Simon, D. T.; Bortolotti, C. A., Organic electrochemical transistor aptasensor for interleukin-6 detection. *ACS Applied Materials & Interfaces* **2023,** *16* (45), 61467–61474.
4. Tanak, A. S.; Muthukumar, S.; Krishnan, S.; Schully, K. L.; Clark, D. V.; Prasad, S., Multiplexed cytokine detection using electrochemical point-of-care sensing device towards rapid sepsis endotyping. *Biosensors and Bioelectronics* **2021,** *171*, 112726.
5. Oh, C.; Park, B.; Li, C.; Maldarelli, C.; Schaefer, J. L.; Datta-Chaudhuri, T.; Bohn, P. W., Electrochemical immunosensing of interleukin-6 in human cerebrospinal fluid and human serum as an early biomarker for traumatic brain injury. *ACS Measurement Science Au* **2021,** *1* (2), 65-73.
6. Aydın, E. B., Highly sensitive impedimetric immunosensor for determination of interleukin 6 as a cancer biomarker by using conjugated polymer containing epoxy side groups modified disposable ITO electrode. *Talanta* **2020,** *215*, 120909.
7. Aydın, E. B.; Aydın, M.; Sezgintürk, M. K., The development of an ultra-sensitive electrochemical immunosensor using a PPyr-NHS functionalized disposable ITO sheet for the detection of interleukin 6 in real human serums. *New Journal of Chemistry* **2020,** *44* (33), 14228-14238.
8. Yang, T.; Wang, S.; Jin, H.; Bao, W.; Huang, S.; Wang, J., An electrochemical impedance sensor for the label-free ultrasensitive detection of interleukin-6 antigen. *Sensors and Actuators B: Chemical* **2013,** *178*, 310-315.
9. Prabhu, K.; Lakshminarayanan, M.; Mohankumar, G.; Ponpandian, N.; Viswanathan, C., Vertically pillared α-Fe2O3 nanorods on carbon yarn as a textile-based stable immunosensor electrode for selective electrochemical sensing of interleukin-6 cancer biomarker. *Sensors and Actuators A: Physical* **2023,** *357*, 114419.
10. Madhu, S.; Han, J. H.; Jeong, C. W.; Choi, J., Sensitive electrochemical sensing platform based on Au nanoflower-integrated carbon fiber for detecting interleukin-6 in human serum. *Analytica Chimica Acta* **2023,** *1238*, 340644.
11. Chu, H.; Hu, X.; Lee, C.-Y.; Zhang, A.; Ye, Y.; Wang, Y.; Chen, Y.; Yan, X.; Wang, X.; Wei, J., A wearable electrochemical fabric for cytokine monitoring. *Biosensors and Bioelectronics* **2023,** *232*, 115301.
12. Tertiş, M.; Ciui, B.; Suciu, M.; Săndulescu, R.; Cristea, C., Label-free electrochemical aptasensor based on gold and polypyrrole nanoparticles for interleukin 6 detection. *Electrochimica Acta* **2017,** *258*, 1208-1218.
13. Cancelliere, R.; Di Tinno, A.; Di Lellis, A. M.; Contini, G.; Micheli, L.; Signori, E., Cost-effective and disposable label-free voltammetric immunosensor for sensitive detection of interleukin-6. *Biosensors and Bioelectronics* **2022,** *213*, 114467.
